# Bivariate genome scans incorporating factor and principal component analyses to identify common genetic components of alcoholism, event-related potential, and electroencephalogram phenotypes

**DOI:** 10.1186/1471-2156-6-S1-S114

**Published:** 2005-12-30

**Authors:** Jing-Ping Lin, Colin Wu

**Affiliations:** 1 Office of Biostatistics Research, National Heart, Lung, and Blood Institute, NIH, 6701 Rockledge Drive, MSC 7938, Bethesda, MD 20892-7938, USA

## Abstract

Genetic components significantly contribute to the susceptibilities of alcoholism and its endophenotypes, such as event-related potential measures and electroencephalogram. An endophenotype is a correlated trait which identifies individuals at risk. Correlated traits could be influenced by shared genes. This study is intended to identify chromosome regions that may harbor common genetic loci contributing to alcoholism, event related potential measures and electroencephalogram. All 143 Collaborative Study on the Genetics of Alcoholism families with 1,614 individuals provided by the Genetic Analysis Workshop 14 were used for the analysis with aldx1 as an alcoholism diagnosis. We carried out factor and principal component analyses on the 12 event-related potentials, then bivariate genome scans on aldx1 and electroencephalogram (ecb21), as well as alcoholism and the principal component scores of the event-related potential measures. A univariate genome scan was also carried out on each trait. Factor and principal component analysis on the event-related potential measures showed that the 4 ttths and 4 ntths belong to one cluster (cluster 1), while the 4 ttdts belonged to another (cluster 2). From each cluster, one principal component was extracted and saved as pc1 (for cluster 1) and pc2 (for cluster 2). The results of genome scans revealed only one chromosome region, chromosome 4 q at about 100 cM, identified by several univariate genome scans including aldx1, ecb21, and pc2, and the evidence of linkage increased significantly in the bivariate genome scans of aldx1 and ecb21 and aldx1 and pc2. Our study suggests that the same quantitative trait locus on the chromosome 4 q region, where *ADH3 *is located, may influence the risk of alcoholism, variations of electroencephalogram, and the 4 ttdts of the event-related potential measures.

## Background

In order to further identify and study genetic loci contributing to alcoholism susceptibility, investigators have recently focus on genome scans on alcoholic endophenotypes, such as event-related potential (ERP) and electroencephalogram (EEG). An endophenotype is a correlated trait with a relatively high heritability. It segregates with the illness in the affected relatives and identifies individuals at risk.

Two correlated traits could be influenced by shared genes. The power to localize the shared genes could be improved by bivariate linkage analysis [1]. However, the technical difficulties for a multivariate linkage analysis increase tremendously when the dimensionality of the correlated traits increases. As a well known dimension-reduction strategy for handling datasets with high-dimensional correlated traits, such as the 12 ERPs in the Collaborative Study on the Genetics of Alcoholism (COGA) dataset, a combination factor analysis and principal component analysis can be used to summarize the variability of the variables into a few principal component scores. Genome scans on these factors may be able to identify common susceptibility loci influencing these correlated traits.

This study is intended to identify common genetic loci contributing to alcoholism and its endophenotypes, EEG and ERPs, through a bivariate genome scan on alcoholism and EEG (ecb21) as well as alcoholism and the factors of the 12 ERPs. Before bivariate genome scans, we carried out univariate genome scans on each trait.

## Methods

### Study subjects

We used all 143 COGA families with 1,614 individuals provided by Genetic Analysis Workshop 14 (GAW14) for the analyses. "aldx1" was used as the alcoholism diagnosis.

### ERP measurements

The ERP data are extracted from the case of the visual oddball experiment for 4 electrode placements, 1 for the far frontal left side channel, 2 for the frontal midline channel, 3 for the central midline channel, and 4 the for parietal midline channel. For the 4 ttths (ttth1 to ttth4) and 4 ttdts (ttdt1 to ttdt4), the data are extracted from the target case. The extracted measures correspond to the 'late' time window, which is set at 300–700 ms following stimulus presentation (bounding the visual P3 event), and the theta band power (3–7 Hz) for the 4 ttths, and the delta band power (1–2.5 Hz) for the 4 ttdts. The 4 ntths (ntth1 to ntth4) contain data extracted from the non-target case. The extracted measures correspond to the 'early' time window, which is set at 100 to 300 ms following the stimulus presentation, and the theta band power (3–7 Hz).

### Factor analysis and principal component analysis

Preliminary examination of the correlation matrix of the 12 ERP variables suggested that these variables could be roughly divided into two clusters, where there were high intra-cluster correlations and low inter-cluster correlations. To verify the fitness of a two-factor model for the ERP variables, we fitted the model *Y *= *μ *+ Λ*f *+ *u*, where *Y *denoted the 12 × 1 vector of the ERP variables, Λ = {*λ*_*ij*_} denoted a 12 × 2 loading matrix, *f *= (*f*_1_,*f*_2_)' denoted the 2 × 1 vector of common factors, and *u *denoted the 12 × 1 vector of unique factors. This model assumed that there were 2 unobserved latent factors, *f*_1 _and *f*_2_, which explained most of the variations of the ERP measurements [2]. The maximum likelihood method with the varimax rotation was used to compute the loading estimates. We then performed a principal component analysis on each of the 2 clusters (i.e., using only the variables within the cluster) [2,3], and obtained the principal component scores pc1 and pc2 from cluster 1 and cluster 2, respectively. For both the factor analysis and the principal component analysis, we assumed that the pedigree members were independent [4] and computed each individual's pc1 and pc2 using the loading estimates. Numerical computations were carried out using the S-plus 6 and the SPSS 12 statistical software packages.

### Heritability estimation and univariate and bivariate genome scan

Variance component linkage analysis implemented in SOLAR (v. 2.1.2) [5] was used for heritability estimation, and univariate and bivariate linkage analyses with two covariates, age at interview and sex. We used a COGA microsatellite marker set with 328 markers and an average genetic spacing about 10 cM for the linkage analyses. For the bivariate linkage analysis, bivariate LOD scores are reported with 1 degree of freedom, which are comparable to the univariate LOD scores.

## Results

The loading matrix computed from the 2-factor model on ERP showed that a) the loading estimates of the 4 ttth ERP variables and the 4 ntth ERP variables were all >0.7 for factor 1 and nearly zero (<0.001) for factor 2; b) the loading estimates of the 4 ttdt ERP variables were (0.326, 0.311, 0.209, 0.255) for factor 1 and (0.215, 0.583, 0.904, 0.736) for factor 2. These results suggested that the 4 ttth ERP and the 4 ntth ERP variables contributed almost exclusively to factor 1, while the 4 ttdt ERP variables contributed almost exclusively to factor 2. These 2 factors explained approximately 63% of the total variation of the ERP measurements. Our principal component analysis showed that pc1, the principal component score computed from the 8 ERP variables in cluster 1, accounted for approximately 80% of the total variation in cluster 1. Similarly, pc2, the principal component score for the 4 ERP variables in cluster 2, accounted for approximately 92% of the total variation in cluster 2. This approach effectively reduced the original 12 correlated ERP variables to two summary scores, pc1 and pc2.

The heritability of aldx1, ecb21, pc1, and pc2 was 0.25 ± 0.07, 0.30 ± 0.05, 0.38 ± 0.06, and 0.31 ± 0.06, respectively, after adjusting for covariates age at interview and sex.

Table 1 displays the maximum multipoint LOD scores (≥ 1.5) based on the univariate and bivariate genome scans, adjusted for age at interview and sex. The univariate genome scan on aldx1 shows a LOD score of 3.14 for chromosome 4 at 96 cM. On the other hand, the bivariate genome scan of aldx1 and ecb21 shows a LOD score of 4.38 for chromosome 4 at 95 cM, and the bivariate genome scan of aldx1 and pc2 shows a LOD score of 4.18 for chromosome 4 at 99 cM. Both the univariate and the bivariate genome scans effectively reveal the same and the only chromosomal area being identified, while the bivariate genome scans provide a stronger evidence of linkage than the univariate genome scans. It is also of interest to note that the bivariate genome scan of aldx1 and pc1 has a LOD score of 3.13 for chromosome 4 at 99 cM, a result virtually the same as a genome scan using aldx1 alone. This result suggests that the contribution provided by pc1 is negligible, as opposed to the contribution provided by pc2.

**Figure 1:**
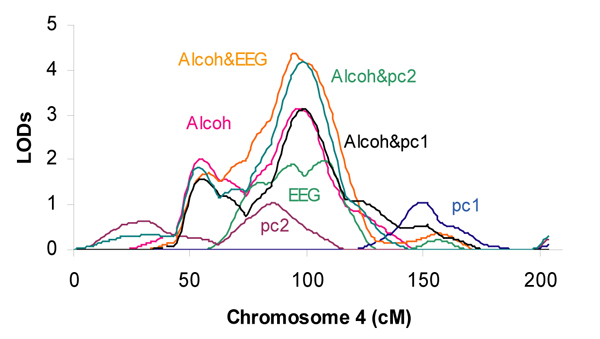
**Multipoint LOD scores on chromosome 4. **X-axis values are centimorgans. Y-axis values are multipoint LOD scores. Linkage analyses were conducted using multivariable residuals, adjusted for age at interview and sex.

Figure 1 displays the maximum multipoint LOD scores on chromosome 4, one region of which was commonly identified by several genome scans.

## Discussion

Our study suggests that the variations of the 4 ttths and the 4 ntths may be mostly controlled by a major factor while the 4 ttdts by another. Both factors have strong genetic components with relatively high heritabilities.

There is one and only one chromosome region, chromosome 4 q about 100 cM, that is identified by several univariate genome scans, aldx1, ecb21 and pc2, and the evidence of linkage was significantly increased in the two bivariate genome scans: aldx1 and ecb21, aldx1 and pc2.

Previous studies have reported significant linkage between alcoholism and this chromosome 4 region near the class I alcohol dehydrogenase locus *ADH3 *[6,7]. Linkage evidence was also found between this chromosome area and EEG results (information provided by GAW14). One study revealed joint consideration of the diagnosis of alcoholism and an ERP (P300, other than the 12 ERPs already discussed) significantly increased the evidence for linkage of those traits to this chromosome area [8].

Through a combination of statistical dimension-reduction techniques (factor and principal component analyses) and bivariate genome scans, our study further suggests that the same quantitative trait locus on the chromosome 4 region, where *ADH3 *is located, may influence the risk of alcoholism, variations of EEG, and the 4 ttdts. The other 8 ERPs may be controlled by other genetic loci.

We also carried out bivariate genome scans on ecb21 and pc1 and ecb21 and pc2 (data not shown). The joint consideration of the traits did not increase the evidence of linkage of those traits to this chromosome 4 area.

It would be interesting to see the results of a simultaneous trivariate genome scan on aldx1, ecb21, and pc2. However, the software we used (SOLAR) does not allow us to carry out this kind of analysis.

## Conclusion

Through factor and principal component analyses on the12 ERP variables, followed by univariate and bivariate genome scans on alcoholism, EEG, and principal component scores of the 12 ERPs, our study suggested that the same quantitative trait locus on the chromosome 4 q region, where *ADH3 *was located, may influence the risk of alcoholism, variations of EEG, and 4 ttdts of the 12 ERPs.

## Abbreviations

COGA: Collaborative Study on the Genetics of Alcoholism

EEG: Electroencephalogram

ERP: Event-related potential

GAW14: Genetic Analysis Workshop 14

## Authors' contributions

J-PL carried out study design, data analysis and drafted the manuscript. CW participated in study design, data analysis, and helped to draft the manuscript.

## Figures and Tables

**Table 1:** Maximum multipoint LOD scores and the chromosomal locations

Trait	Chromosome	Location (cM)	LOD
aldx1	3	135	2.63
	4	55	2.00
	4	96	3.14
			
ecb21	4	108	1.96
	8	41	1.71
			
pc1	2	252	1.60
			
pc2	1	62	1.79
	4	85	1.05^a^
	11	110	1.70
	16	68	1.68
	18	54	2.30
			
aldx1&ecb21	4	95	4.38
			
aldx1&pc1	3	134	1.85
	4	99	3.13
			
aldx1&pc2	1	64	1.65
	3	136	1.98
	4	53	1.82
	4	99	4.18
	18	82	2.00

^a^Though the LOD score here is less than 1.5, the bivariate LOD score on aldx1&pc2 in this area showed a significant increase compared with that of the univariate on aldx1 alone. Therefore, the LOD score on this area of the univariate analysis on pc2 is listed.

## References

[B1] Almasy L, Dyer TD, Blangero J (1997). Bivariate quantitative trait linkage analysis: pleiotropy versus co-incident linkages. Genet Epidemiol.

[B2] Jolliffe IT (1986). Principal Component Analysis.

[B3] Muller KE (1982). Understanding canonical correlation through the general linear model and principal components. Am Statistician.

[B4] Moser KL, Jedrey CM, Conti D, Schick JH, Gray-McGuire C, Nath SK, Daley D, Olson JM (2001). Comparison of three methods for obtaining principal components from family data in genetic analysis of complex disease. Genet Epidemiol.

[B5] Almasy L, Blangero J (1998). Multipoint quantitative-trait linkage analysis in general pedigrees. Am J Hum Genet.

[B6] Reich T, Edenberg HJ, Goate A, Williams JT, Rice JP, Van Eerdewegh P, Foroud T, Hesselbrock V, Schuckit MA, Bucholz K, Porjesz B, Li TK, Conneally PM, Nurnberger JI, Tischfield JA, Crowe RR, Cloninger CR, Wu W, Shears S, Carr K, Crose C, Willig C, Begleiter H (1998). Genome-wide search for genes affecting the risk for alcohol dependence. Am J Med Genet.

[B7] Long JC, Knowler WC, Hanson RL, Robin RW, Urbanek M, Moore E, Bennett PH, Goldman D (1998). Evidence for genetic linkage to alcohol dependence on chromosomes 4 and 11 from an autosome-wide scan in an American Indian population. Am J Med Genet.

[B8] Williams JT, Begleiter H, Porjesz B, Edenberg HJ, Foroud T, Reich T, Goate A, Van Eerdewegh P, Almasy L, Blangero J (1999). Joint multipoint linkage analysis of multivariate qualitative and quantitative traits. II. Alcoholism and event-related potentials. Am J Hum Genet.

